# L-type calcium channel blocker increases VEGF concentrations in retinal cells and human serum

**DOI:** 10.1371/journal.pone.0284364

**Published:** 2023-04-13

**Authors:** Anmol Kumar, Stefan Mutter, Erika B. Parente, Valma Harjutsalo, Raija Lithovius, Sinnakaruppan Mathavan, Markku Lehto, Timo P. Hiltunen, Kimmo K. Kontula, Per-Henrik Groop

**Affiliations:** 1 Folkhälsan Institute of Genetics, Folkhälsan Research Center, Helsinki, Finland; 2 Research Program for Clinical and Molecular Metabolism, Faculty of Medicine, University of Helsinki, Helsinki, Finland; 3 Department of Nephrology, University of Helsinki and Helsinki University Hospital, Helsinki, Finland; 4 Vision Research Foundation, Sankara Nethralaya, Chennai, India; 5 Department of Medicine, University of Helsinki & Helsinki University Hospital, Helsinki, Finland; 6 Department of Diabetes, Central Clinical School, Monash University, Melbourne, Australia; SRM Medical College Hospital and Research Centre, INDIA

## Abstract

**Objective:**

Vascular endothelial growth factor (VEGF) plays a key role in diabetic retinopathy (DR). Previously, we have reported an association between mutations in a gene coding for the L-type calcium channel subunit, VEGF and DR. L-type calcium channel blockers (LTCCBs) have been widely used as antihypertensive medication (AHM), but their association with VEGF and DR is still unclear. Therefore, we explored the effect of LTCCBs compared to other AHMs on VEGF concentrations in retinal cells and human serum. Furthermore, we evaluated the association between the use of LTCCBs and the risk of severe diabetic eye disease (SDED).

**Research design and methods:**

Müller cells (MIO-M1) were cultured as per recommended protocol and treated with LTCCBs and other AHMs. VEGF secreted from cells were collected at 24 hours intervals. In an interventional study, 39 individuals received LTCCBs or other AHM for four weeks with a four-week wash-out placebo period between treatments. VEGF was measured during the medication and placebo periods. Finally, we evaluated the risk of SDED associated with LTCCB usage in 192 individuals from the FinnDiane Study in an observational setting.

**Results:**

In the cell cultures, the medium VEGF concentration increased time-dependently after amlodipine (P<0.01) treatment, but not after losartan (P>0.01), or lisinopril (P>0.01). Amlodipine, but no other AHM, increased the serum VEGF concentration (P<0.05) during the interventional clinical study. The usage of LTCCB was not associated with the risk of SDED in the observational study.

**Conclusions:**

LTCCB increases VEGF concentrations in retinal cells and human serum. However, the usage of LTCCBs does not appear to be associated with SDED in adults with type 1 diabetes.

## Introduction

Diabetic retinopathy (DR) is a devastating complication among individuals with diabetes and may lead to permanent vision loss. Given the increasing number of individuals with diabetes worldwide, the prevalence of DR is expected to rise significantly in the coming years [1]. Hyperpermeability, hypoperfusion, and neo-angiogenesis of the intraocular microvasculature are key hallmarks of DR and will ultimately lead to anatomical and pathophysiological alterations [2].

Vascular endothelial growth factor (VEGF) is a well-known angiogenic factor in the eye, and is also a major pharmacological target for the treatment of severe and proliferative diabetic retinopathy (PDR) [3, 4]. Although, intraocular injections of anti-VEGF antibodies have emerged as a novel and effective pharmacological treatment, long-term efficacy and systemic safety data are lacking, stressing the need to explore alternative pathways and molecular targets for intervention [5]. Recently, we reported mutations in the *CACNB2* gene encoding the L-type calcium channel’s β-subunit. These mutations contributed to the severity of PDR. Furthermore, after down-regulating the β-subunit coding *CACNB2* gene by RNA interference, the concentration of VEGF was significantly reduced in retinal cells [6].

Notably, a network meta-analysis showed that renin-angiotensin-aldosterone system inhibitors (RAASi) were associated with reduced risk of progression and increased the possibility of regression of DR [7]. However, although the calcium channel blockers seemed to worsen the outcome compared to placebo, it was not statistically significant [7]. L-type calcium channel blockers (LTCCB) such as amlodipine have been widely prescribed to treat arterial hypertension as monotherapy or in combination with other classes of antihypertensive medications (AHM) including angiotensin-converting enzyme inhibitors (ACEi) such as lisinopril, angiotensin II receptor blockers (ARB) such as losartan, β blockers such as bisoprolol and diuretics such as hydrochlorothiazide [8, 9]. However, the relationship between LTCCB and DR is still poorly understood.

Therefore, in this work, we aimed to explore the effect of LTCCBs in comparison to other AHMs on the VEGF concentrations in retinal cell culture and serum of human subjects. We used human retina-derived Müller cells for *in vitro* experiments as these cells are a crucial source of VEGF in the pathogenesis of DR [10, 11]. In our experiments, we evaluated the effects of amlodipine, a commonly used LTCCB, alongside drugs of other subclasses of AHMs on the serum VEGF concentrations in *in vitro* experiments and in a clinical trial setting (The GENRES Study). These drugs were chosen as each of them represents a typical medication of the respective antihypertensive subclass, each has a favourable profile in terms of effectiveness versus side effects and each has been widely used as an antihypertensive agent in clinical and experimental settings. Finally, we evaluated the association between the use of LTCCB and the risk of severe diabetic eye disease (SDED) in adults with type 1 diabetes from the Finnish Diabetic Nephropathy Study (FinnDiane) cohort.

## Research design and methods

### Retinal cell culture study

Müller glia or Müller cell lines, MIO-M1 were obtained from Prof. Astrid Limb [12]. MIO-MI cells were a gift from Prof. Astrid Limb, Ocular Repair and Regeneration Biology Unit, Departments of Cell Biology and Pathology, Institute of Ophthalmology and Moorfields Eye Hospital, London, United Kingdom; Prof. Limb has ethics committee approvals from local health authority, eyes consented for research were obtained from Moorfields Hospital Eye Bank, London, United Kingdom to generate MIO-M1 cells in her laboratory. Cells were grown in Dulbecco’s Modified Eagle Medium (41965–039, Gibco/life technology), 10% Fetal Bovine Serum (10270106, Gibco/life technology), Penicillin-Streptomycin (15140122, Gibco/life technology) and Normocin™ (ant-nr-1, InvivoGen) in a humidified 5% CO_2_ cell culture incubator at +37°C temperature. Cells were plated in 6-well plates (140675, Nunc™) with seeding density (~10^6^ cells/well). Three medications—amlodipine besylate (A5605, Sigma-Aldrich), losartan potassium (phr1602-1g, Sigma-Aldrich) and lisinopril (phr1143-1g, Sigma-Aldrich) were dissolved in solvents as per the manufacturer’s recommendation. Stock solution of 17.6 mM in DMSO (dimethyl sulfoxide) was prepared for amlodipine, 20 mM stock solution in 1XPBS for losartan and 25 mM stock solution for lisinopril in 1X PBS based on their solubility characteristics and chemical properties. The final working concentration (1–10 μM) of the drugs were achieved by further diluting the stock solution in DMEM supplemented with 0.02% bovine serum albumin (BSA) and antibiotics (Penicillin-Streptomycin- Normocin™). The medications and the dimethyl sulfoxide (DMSO)/1X Phosphate-buffered saline (PBS) control (the solvent used to dissolve the medication) were added in equal volume to the cell culture medium. The cell culture medium containing medications and control in equal volume were changed every 24 hours until the end of the experiment. Cell culture medium was collected and centrifuged at +8°C temperature at 3000 rpm for 5 minutes to remove cell debris, and the resulting supernatants were collected and stored in a -80°C freezer for further analysis.

### Interventional clinical study

The GENRES Study is a randomized, double-blind, placebo-controlled, crossover study, which included Finnish men with moderate hypertension but without drug-treated diabetes. A total of 313 hypertensive Finnish men (aged 35 to 60 years) were initially recruited. The clinical study was completed by 228 subjects and blood serum samples (collected in the morning under non-fasting conditions and stored at -80°C without any incidental thawing) were available for VEGF measurement assays from 39 subjects. The GENRES Study was approved by the Ethics Committee of Helsinki University Central Hospital. The clinical part of the study was carried out in accordance with the Declaration of Helsinki and Guidelines for Good Clinical Practice. All subjects gave a signed informed consent prior to study activities. The study is registered at ClinicalTrials.gov (NCT03276598). The detailed protocol can be found in a previous publication [13]. In brief, the GENRES study started with a four-week run-in placebo period, before which the participants discontinued their previous antihypertensive medication, in case they were on medication. Then, participants were randomized into different sequences of four-week treatment periods taking one of the AHM (amlodipine, bisoprolol, hydrochlorothiazide, losartan) at a time. Thus, each participant underwent four separate monotherapy periods, separated by four-week placebo periods [13]. Serum VEGF concentration was measured at eight different time points, at the end of the four placebo and the four drug treatment periods. Notably, the serum at the end of the first placebo period was not included in the analysis, since the blood sample was taken after an overnight fast as opposed to the other blood samples, which were taken in the non-fasting state.

### VEGF measurements

VEGF measurements were performed double-blinded in serum and cell culture media using the Human VEGF Quantikine ELISA Kit (DVE00 & SVE00, R&D systems/bio-techne) according to the manufacturer’s protocol.

### Observational cohort study

The outcome was SDED, defined as a composite outcome including proliferative diabetic retinopathy (PDR), initiation of laser treatment or anti-VEGF therapy, diabetic maculopathy, vitreous hemorrhage and vitrectomy identified from the Finnish Care Register for Health Care until the end of 2015.

Information on purchases of AHMs was obtained from the Finnish Drug Prescription Register (maintained by the National Social Insurance Institution since 1994, which contains information on all prescribed, purchased, and reimbursed medications in outpatient care) until the end of 2015. Medications were coded according to the Anatomic Therapeutic Chemical (ATC) classification system. The renin-angiotensin-aldosterone system inhibitors (RAASi) were identified by an ATC code starting with C09 including both ACEis and ARBs. To identify individuals on RAASi monotherapy, we excluded individuals with a prescription record of combination products that contained other AHMs in the same pill (coded with C09B and C09D) or with a prescription for any other AHMs prior to the baseline or during the follow-up. LTCCBs in the register included the following ATC codes: C07FB02, C07FB03, C08CA01, C08CA02, C08CA03, C08CA05, C08CA07, C08CA10, C08CA13, C09BB02, C09BB04, and C09BB05. Refill adherences were calculated using the so-called proportion of days covered method with a prescription refill interval of 90 days.

For this longitudinal study, 192 individuals were selected from the FinnDiane study, which is an ongoing, nationwide, multicenter cohort of adults with type 1 diabetes in Finland [14, 15]. Type 1 diabetes was defined as age of diabetes onset below 40 years and requiring insulin within the first year after diagnosis. Currently, more than 5,400 individuals have undergone a detailed clinical examination at their first FinnDiane visit. We excluded 1,933 individuals due to SDED at baseline. These data (history of laser treatment) were registered based on medical records at baseline and obtained by the attending physician or nurse using a standardized form. The selection criteria for the remaining 3,468 individuals can be found in the S1 Fig. First, we required the study visit to take place at least half a year before the end of the prescription records at the end of 2015 and no SDED in the first half year of follow-up. As shown in S1 Fig, our cohort is made up of two groups: those on LTCCB plus RAASi and other AHM and a control group of those on RAASi as monotherapy. In both groups individuals had been on RAASi therapy at baseline, defined as either already being on RAASi prior to the study visit or starting RAASi therapy no longer than half a year after the visit. We further required at least 2 purchases of RAASi during the follow-up. The same selection criteria were applied for LTCCB in the group LTCCB plus RAASi and other AHM. In this latter group, individuals were allowed to use other AHMs, because, according to the hypertension guidelines, LTCCBs are usually considered as the second or third line drug after RAASi [16, 17]. Due to the strict adherence to the guidelines in Finland, we did not find enough individuals using LTCCB as monotherapy (n = 11) to be able to investigate them as a separate group. All subjects participating in the study have been informed about the study and have given their informed consent before participation. The participants also consented to obtain and utilize the health registry data. We have permission Dnro THL/1519/5.05.00/2013 for the data from the Finnish Care Register for Health Care and Dnro Kela/22/522/2013 for the Finnish Drug Prescription Register data and the updated permission Dnro THL/207/14.02.00/2021 for both registers. The study follows the General Data Protection Regulation (EU GDPR). The study protocol was approved by the Ethical Committee of Helsinki and Uusimaa Hospital District (HUS/3318/2018).

### Statistical analysis

Cell culture data were analyzed using nonparametric Mann-Whitney U test (S1 Table). A Bonferroni corrected p-value below 0.0125 was considered significant in the cell culture experiments. Fold changes in the VEGF concentration are calculated by dividing the concentration at a timepoint by the mean control concentration. For data collection for the statistical analysis, *in vitro* experiments were repeated under similar experimental settings with 2–3 technical repeats/cell culture wells for each treatments.

In the analysis of the clinical study (The GENRES Study) results, the VEGF concentrations after the drug treatment periods were compared pairwise with the baseline VEGF concentrations (the mean values after the three placebo periods) for each subject. Since VEGF concentrations at baseline and changes after the drug treatment periods were non-normally distributed, the nonparametric Wilcoxon signed ranks test was used to test the statistical significance of the drug-induced changes. The primary target variable in these analyses was the change caused by amlodipine treatment and the other drug treatments were analysed for their drug-specificity of the effect. Therefore, no correction for multiple comparisons was applied and a p-value below 0.05 was considered statistically significant.

In the observational study, a multivariable logistic regression analysis was used to study the associations between the usage of LTCCB plus RAASi and AHM (n = 62) and the incidence of SDED as the outcome. Since RAASi usage has shown an association with reduced risk of progression and increased possibility of regression of DR [7], first, we performed a logistic regression using the adherence to RAASi usage as the independent variable and the incidence of SDED (n = 130) as the outcome. Since the adherence to RAASi usage was associated with the outcome, this variable was included as a covariate in the logistic regression model for the analysis of LTCCB plus RAASi and AHM and incident SDED alongside the presence of any retinopathy other than SDED at baseline and clinical baseline variables that were significantly different between those that developed SDED and those that did not (Table 1). Therefore, the full set of covariates in the multivariable model was age, age of diabetes onset, presence of albuminuria, HbA1c, adherence to RAASi treatment during follow-up, other antihypertensive medication on top of LTCCB plus RAASi during follow-up and the presence of any retinopathy other than SDED at baseline. For continuous variables, p-values were calculated using t-tests for normally distributed variables and Mann-Whitney U-tests for non-normally distributed variables. For categorical variables, we used the χ2-test. P-value below 0.05 was considered significant. All analyses on observational data were performed in R version 3.6 [18].

**Table 1:** Characteristics of individuals with type 1 diabetes participating in the observational study.

	No SDED	Incident SDED	*P-value*
N	122	70	
LTCCB (%)	32.8	31.4	0.97
Other AHM (%)	31.1	22.9	0.29
Women (%)	41.0	38.6	0.86
Age (years)	42.82 ± 10.65	36.30 ± 11.93	<0.001
Age of diabetes onset (years)	18.34 ± 8.10	12.92 ± 7.72	<0.001
Diabetes duration (years)	24.50 ± 11.75	23.38 ± 9.71	0.50
Systolic blood pressure (mmHg)	141 ± 18	137 ± 17	0.10
Diastolic blood pressure (mmHg)))) ´)mmHg)mmHg(mmHg)	80 ± 10	81 ± 10	0.43
Total cholesterol (mmol/L)	4.00 ± 1.12	4.89 ± 1.04	0.93
Triglycerides (mmol/L)	0.03 (0.69, 1.33)	1.08 (0.81, 1.40)	0.08
eGFR (mL/min/1.73m^2^)	93 ± 28	96 ± 29	0.43
Albuminuria (%)	31.1	70.0	<0.001
HbA_1c_ (%)	8.2 ± 1.3	9.3 ± 2.0	<0.001
HbA_1c_ (mmol/mol)	66 ± 14	78 ± 22	<0.001
RAASi before baseline (years)	3.04 ± 2.77	2.79 ± 2.31	0.53
Adherence to RAASi (%)	74.94 ± 17.20	70.05 ± 20.58	0.08
Any retinopathy except SDED (%)	52.6	67.7	0.07

Data are shown as mean ± SD or median (interquartile range). SDED, severe diabetic eye disease; LTCCB, L-type calcium channel blocker; RAASi, renin-angiotensin-aldosterone system inhibitors; AHM, antihypertensive medication other than LTCCB or RAASi, eGFR, estimated glomerular filtration rate; HbA_1c_, glycated haemoglobin A_1c_.

## Results

### Retinal cell culture study

**Fig 1:**
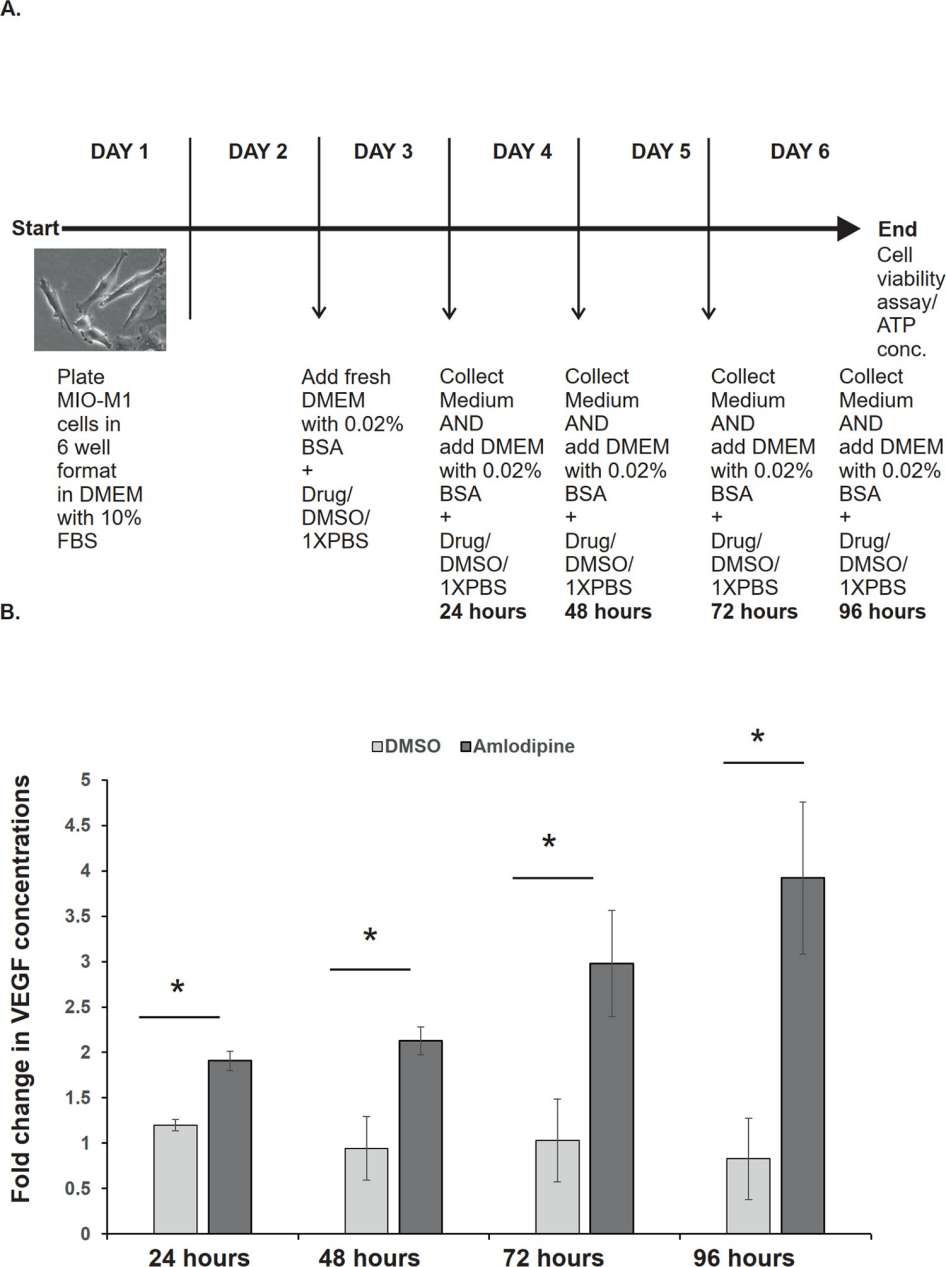
A: Schematic diagram showing the experimental setup and the sample collection timeline. B: Vertical bars represent the mean and the standard deviation of fold changes in the VEGF concentrations in the MIO-M1 cell culture medium at different time points (24 hours, 48 hours, 72 hours and 96 hours) after treatment with amlodipine (10 μM) vs control. Fold changes in the VEGF concentration are calculated by dividing the concentration at a timepoint by the mean control concentration of 125.8765 pg/ml. *P-value = 0.007937.

After treating the MIO-M1 cells with 10 μM amlodipine at 24, 48, 72 and 96 hours, the treatment increased the secreted VEGF protein in the medium compared to each respective control treatment (Fig 1B).

In contrast, the secreted VEGF protein in the medium did not change after lisinopril or losartan treatments at any time points compared to each respective control treatment (S2A and S2B Fig).

Additionally, we tested lower concentrations of amlodipine (1 μM and 5 μM), but they did not affect the VEGF concentration in the medium of the cell culture at 24, 48, 72 and 96 hours (S3 Fig). Finally, we performed cell viability tests at the end of our experiment (96 hours). Amlodipine (10 μM) reduced cell proliferation, whereas losartan (10 μM) and lisinopril (10 μM) did not affect the cell viability or proliferation compared to the control (DMSO/1XPBS) (S4A and S4B Fig).

### Interventional clinical study

**Fig 2:**
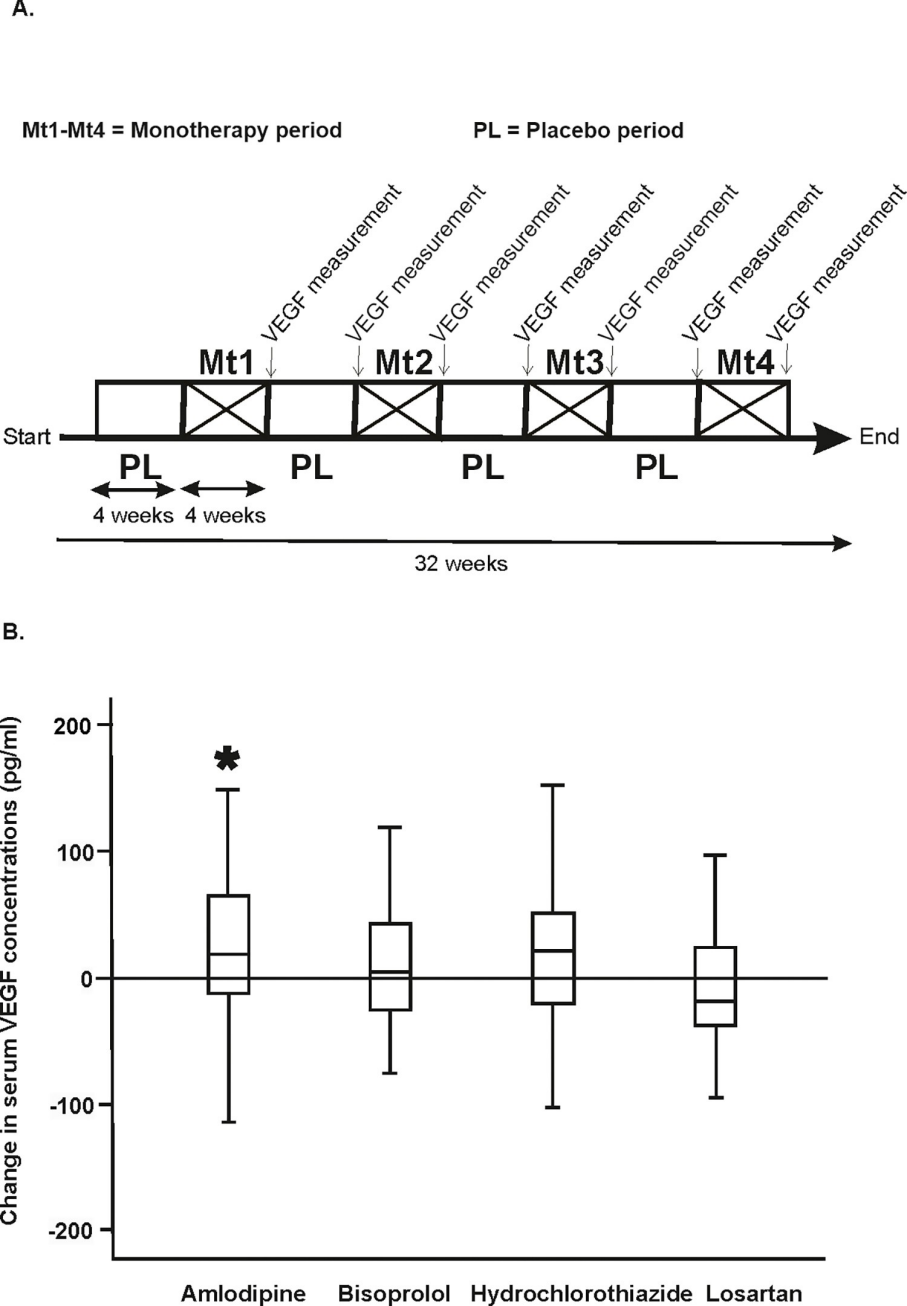
A: Schematic diagram showing the interventional clinical study design and the sample collection timeline. B: Median and interquartile range changes in the serum VEGF concentration after 4 weeks of daily monotherapies with amlodipine (5 mg), bisoprolol (5 mg), hydrochlorothiazide (25 mg) or losartan (50 mg) compared to the wash-out phases. The nonparametric Wilcoxon signed ranks test was used to test the statistical significance of the drug-induced changes in serum VEGF. *P-value < 0.05 denotes statistical significance.

During the placebo periods, the median serum VEGF concentration was 277 pg/mL in 39 men with hypertension. During the LTCCB treatment period, there was a median increase of 18 pg/ml in the serum VEGF concentration (p-value = 0.03). Treatment with hydrochlorothiazide induced a nonsignificant increase (p-value = 0.09) while treatment with losartan or bisoprolol caused no significant changes in the VEGF concentration (Fig 2B).

### Observational study

During a mean follow up of 8.6 ± 5.7 years, 70 events of SDED occurred. Individuals who developed SDED had a younger age at onset of diabetes and higher glycated hemoglobin (HbA_1c_) at baseline than those that did not develop SDED. The clinical characteristics of the individuals at baseline, according to the incidence of SDED, are shown in Table 1. The higher refill adherence to RAASi was associated with a lower risk of SDED (OR 0.97, 95% CI ☯0.95–0.99], p-value = 0.005) in an unadjusted model. The use of LTCCB plus RAASi and other AHM was neither associated with increased odds of SDED (OR 0.94, 95% CI [0.50–1.77], p-value = 0.85) in the unadjusted model nor in the adjusted model (OR 6.12, 95% CI [0.88–42.45], p-value = 0.07) (S2 Table).

## Discussion

In the present study, we showed that amlodipine, an LTCCB, increases the secretion of VEGF *in vitro* by cells of human retinal origin, in a time-dependent manner. In addition, we showed that four weeks of amlodipine usage also increases the serum VEGF concentration in individuals with arterial hypertension. However, in our observational study, the use of LTCCB was not associated with an increased risk of SDED in adults with type 1 diabetes.

Our observations are supported by a previous report showing that nifedipine, a first-generation LTCCB, induces VEGF secretion in human coronary artery smooth muscle cells (HCSMCs) [19]. We used 24–96 hours of treatment in contrast to 18–24 hours in previous *in vitro* studies [19, 20]. This prolonged *in vitro* treatment may better represent a chronic pharmacological intervention [19]. Additionally, we observed an anti-proliferative effect of amlodipine on MIO-M1 cells, similar to what has been shown with nifedipine on HCSMCs [19]. Another study has reported no effect of amlodipine on VEGF secretion by HCSMCs. However, the shorter treatment duration and lower medication dosage (5 μM) used in that study may explain the observed differences compared to our results [20]. To reinforce this hypothesis, we also tested 1 μM and 5 μM of amlodipine on MIO-M1 cells, and we did not find any changes in the VEGF concentration at any time of the treatment (24–96 hours). Importantly, DR disrupts the blood-retinal barrier increasing perfusion of medications into the ocular compartment, thus altering intraocular pharmacokinetics and diffusion of medication molecules [21, 22]. This may increase the diffusion of L-type calcium channel blockers from the systemic circulation into the ocular compartments in individuals with DR and thereby elevating their intraocular concentrations. Our data from the interventional clinical study show that the serum VEGF concentration increases after four weeks of amlodipine treatment (5 mg/day). Although a previous study reported a positive association between serum and vitreous VEGF levels [23, 24], we are not able to conclude from our results whether an elevated serum VEGF concentration also translates into an increased VEGF concentration in the vitreous humour. Furthermore, it is not possible to ensure that the increased serum VEGF concentration after four weeks of amlodipine usage will persist after years of treatment. Further *in vivo* experiments using animal models are needed to clarify these questions, since collecting vitreous samples from human subjects is challenging.

The observational study showed that long-term usage of LTCCB plus RAASi and other AHM was not associated with SDED. We are not sure whether the lack of association is due to the combination of LTCCB with RAASi, since RAASi have been linked to lower risk of DR [7, 9]. However, we included refill adherence to RAASi therapy as a covariate in our models. In our study, it was not possible to evaluate the risk of SDED related to the use of LTCCB as monotherapy, because the number of individuals using LTCCB without other medication in the FinnDiane cohort was too small. Indeed, this is aligned with the hypertension treatment guidelines in people with diabetes [16, 17] in which the calcium channel blockers are not the first-line choice and are commonly prescribed in association with RAASi as second or third-line therapy. Although we did not find an association between the use of LTCCB and SDED, we found that a higher adherence to RAASi was associated with lower odds of SDED, similar to what has been previously shown [7].

The major strength of the current study is the *in vitro* experiments with retina-derived Müller cells showing for the first time a time-dependent effect of LTCCB on VEGF concentrations. Additionally, we validated these *in vitro* findings using a unique interventional clinical trial that showed an increase in serum VEGF after four weeks of exposure to LTCCB. The main drawback of our study is that we were not able to measure VEGF in the vitreous humour to evaluate the correlation between the serum and the vitreous VEGF concentrations. Furthermore, despite the inclusion of one of the largest cohorts of individuals with type 1 diabetes (the FinnDiane Study), there was not a large enough number of individuals using LTCCB as monotherapy in order to be able to explore a potential adverse association between the use of LTCCB and SDED.

## Conclusions

Our results show that LTCCB increases VEGF concentrations in retinal origin cells and in human serum, but its usage in combination with RAASi and other AHM does not seem to be associated with SDED in adults with type 1 diabetes in an observational setting. Further studies including a larger sample of individuals with other types of diabetes using LTCCB as monotherapy, as well as studies with animal models to measure the VEGF in the vitreous humour are warranted to evaluate the impact of chronic use of LTCCBs on the development and progression of DR.

## Supporting information

S1 Fig The selection criteria for observational study cohort. Schematic diagram depicting selection criteria in the observational study with FinnDiane cohort.
(TIF)

S2 Fig **A.** Effects on VEGF concentrations in the MIO-M1 cells after treatment with losartan. Vertical bars represent the mean and the standard deviation of fold changes in the VEGF concentrations in the MIO-M1 cell culture medium at different time points (24 hours, 48 hours, 72 hours and 96 hours) after treatment with losartan (10 μM) vs control. Fold changes are calculate by dividing a concentration at a time point by the mean control concentration of 92.04108 pg/ml. NS = Not Significant (Mann-Whitney U test). **B.** Effects on VEGF concentrations in the MIO-M1 cells after treatment with lisinopril. Vertical bars represent the mean and the standard deviation of fold changes in the VEGF concentrations in the MIO-M1 cell culture medium at different time points (24 hours, 48 hours, 72 hours and 96 hours) after treatment with lisinopril (10 μM) vs control. Fold changes are calculate by dividing a concentration at a time point by the mean control concentration of 100.1139 pg/ml. NS = Not Significant (Mann-Whitney U test).
(TIF)

S3 Fig Dose-response determination for amlodipine concentrations and time points to measure VEGF in cell culture media. Dose-response (1, 5 and 10 μM) pilot experiment for determination of effects of amlodipine on VEGF secretion in MIO-M1 cells and to find out an optimal experimental setting for amlodipine concentrations and time points to measure VEGF in cell culture media. Values for VEGF concentrations are calculated as mean of three technical repeats for every treatment from a single experiment.
(TIF)

S4 Fig **A.** MIO-M1 cells viability determination. Survival of MIO-M1 cells at end of drug treatment (96 hours), based on intracellular ATP concentration. Survival mean percentages are calculated from two independent experiments. Vertical lines represent standard deviation. **B.** MIO-M1 cells viability, DMSO vs 1xPBS treatment. Luminescence [quantified as Relative Light Units (RLU)] based assay to measure intracellular ATP indicating general cell health. Our data show no statistically significant difference in the intracellular ATP concentration between DMSO and 1xPBS in MIO-M1 cells; 96 hours post treatment (End of experiment). Vertical lines represent standard deviation.
(TIF)

S1 Table Detailed cell culture experimental data and statistics. Detailed information about statistical analysis and calculations with cell culture experimental data.
(XLSX)

S2 Table Additional information from observational study. Detailed information about statistical analysis and models used in observational study.
(XLSX)

S1 Appendix FinnDiane study centers. List of FinnDiane study centers across Finland.
(DOCX)
