# Supplementary figures and images for: L-type calcium channel blocker increases VEGF concentrations in retinal cells and human serum
Source: PLoS One. 2023 Apr 13;18(4):e0284364. doi: 10.1371/journal.pone.0284364 (PMC10101440; doi:10.1371/journal.pone.0284364)

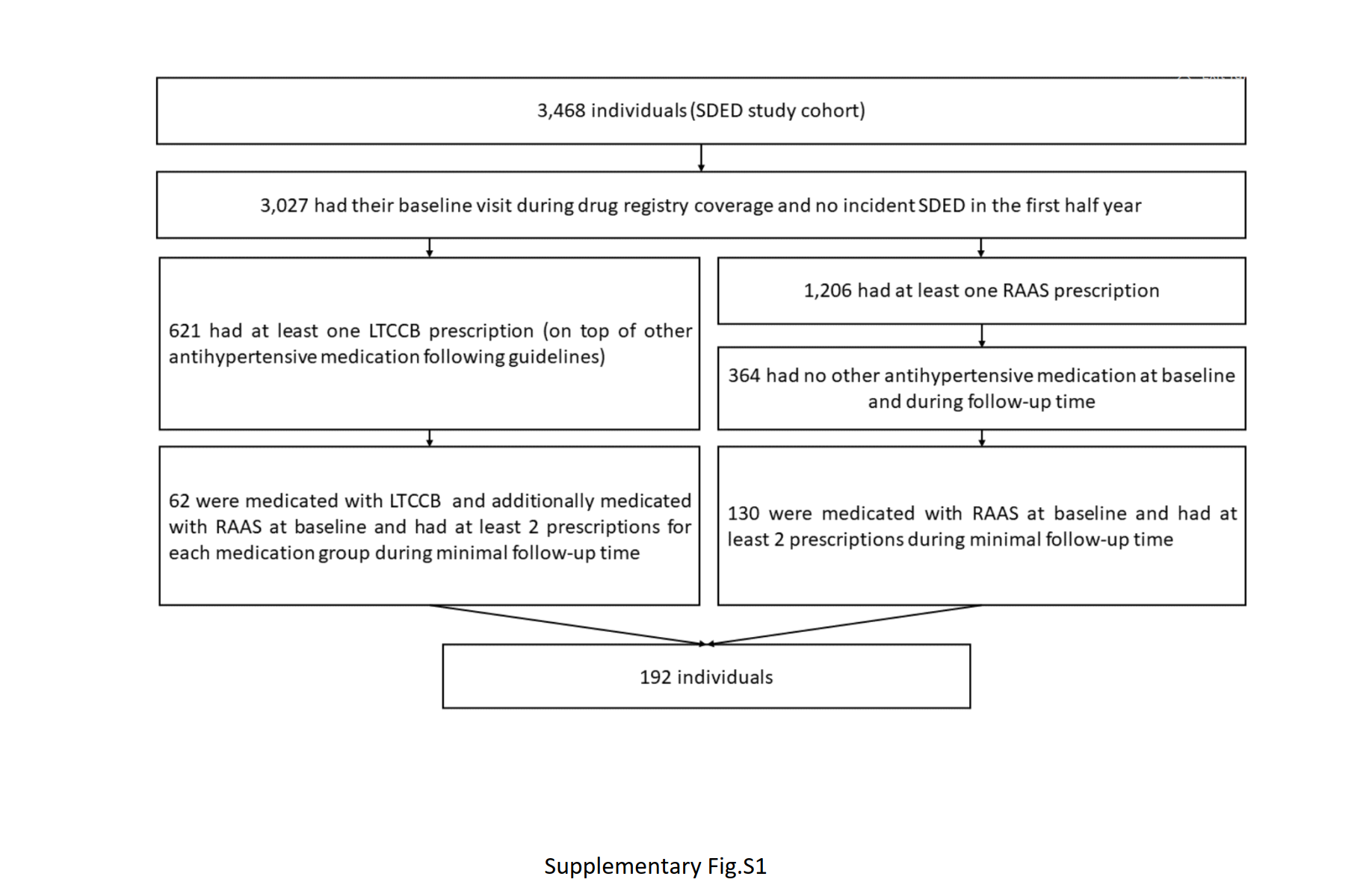

Supplement: S1 Fig — Schematic diagram depicting selection criteria in the observational study with FinnDiane cohort. (TIF) [file pone.0284364.s001.tif]

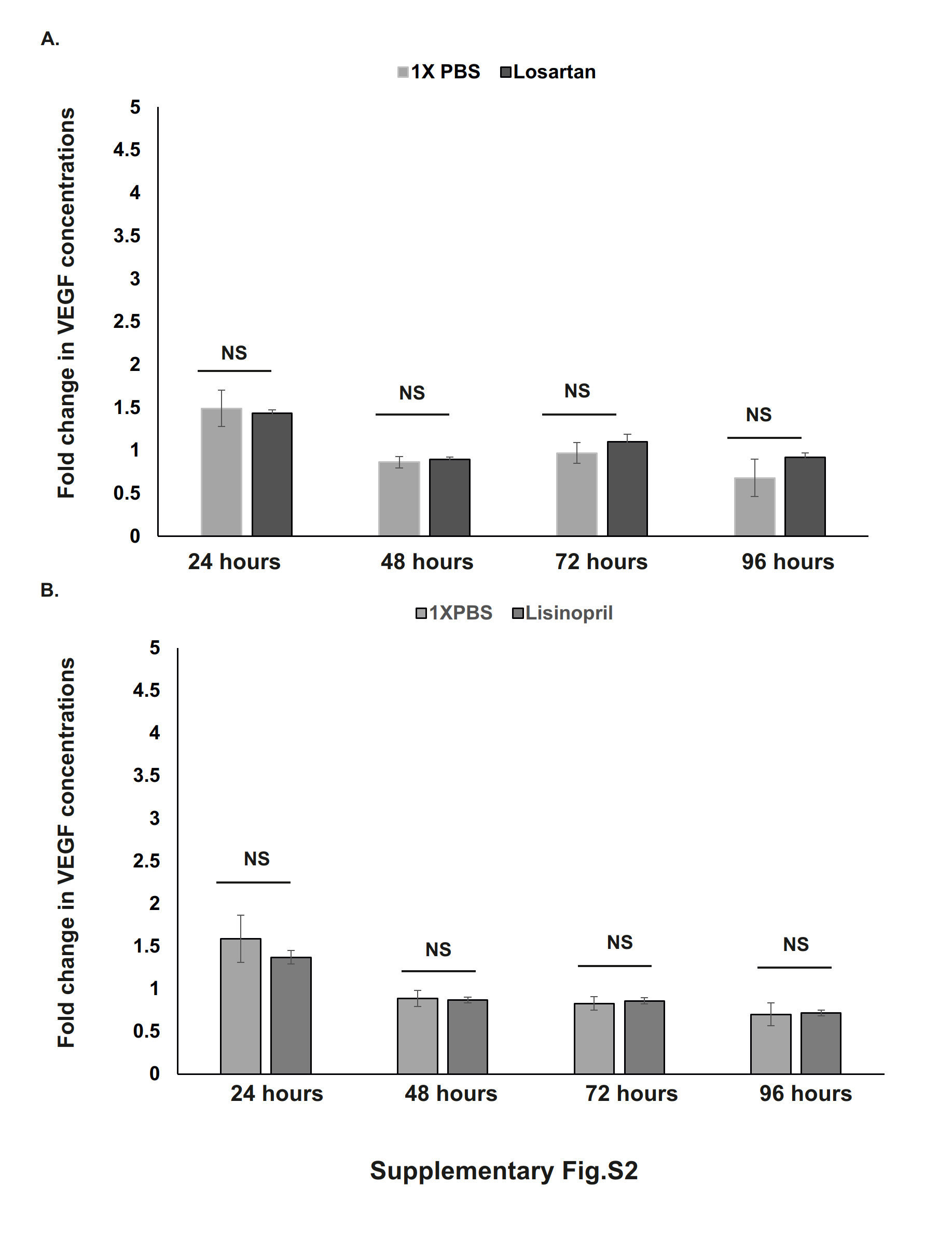

Supplement: S2 Fig — A. Effects on VEGF concentrations in the MIO-M1 cells after treatment with losartan. Vertical bars represent the mean and the standard deviation of fold changes in the VEGF concentrations in the MIO-M1 cell culture medium at different time points (24 hours, 48 hours, 72 hours and 96 hours) after treatment with losartan (10 μM) vs control. Fold changes are calculate by dividing a concentration at a time point by the mean control concentration of 92.04108 pg/ml. NS = Not Significant (Mann-Whitney U test). B. Effects on VEGF concentrations in the MIO-M1 cells after treatment with lisinopril. Vertical bars represent the mean and the standard deviation of fold changes in the VEGF concentrations in the MIO-M1 cell culture medium at different time points (24 hours, 48 hours, 72 hours and 96 hours) after treatment with lisinopril (10 μM) vs control. Fold changes are calculate by dividing a concentration at a time point by the mean control concentration of 100.1139 pg/ml. NS = Not Significant (Mann-Whitney U test). (TIF) [file pone.0284364.s002.tif]

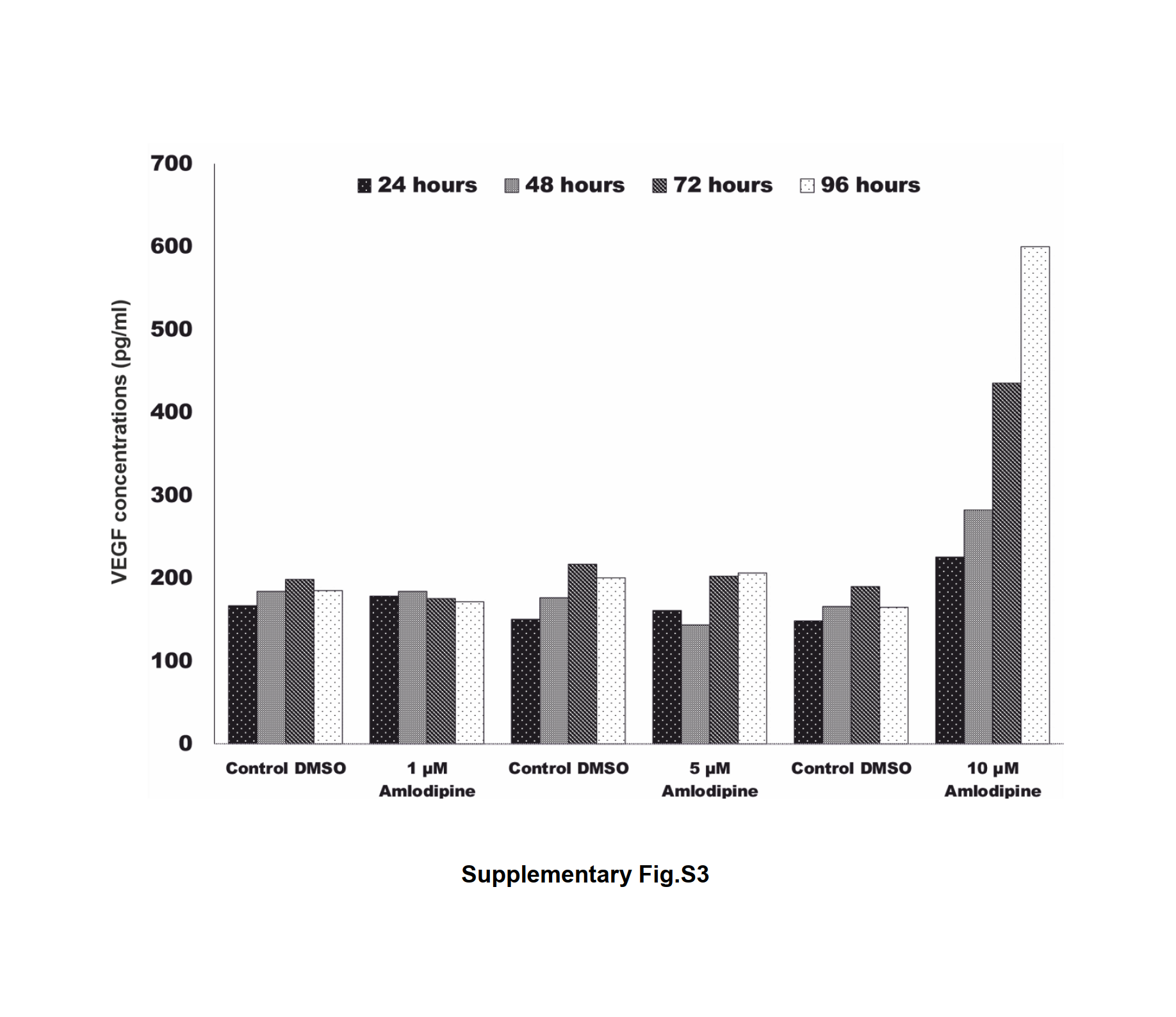

Supplement: S3 Fig — Dose-response (1, 5 and 10 μM) pilot experiment for determination of effects of amlodipine on VEGF secretion in MIO-M1 cells and to find out an optimal experimental setting for amlodipine concentrations and time points to measure VEGF in cell culture media. Values for VEGF concentrations are calculated as mean of three technical repeats for every treatment from a single experiment. (TIF) [file pone.0284364.s003.tif]

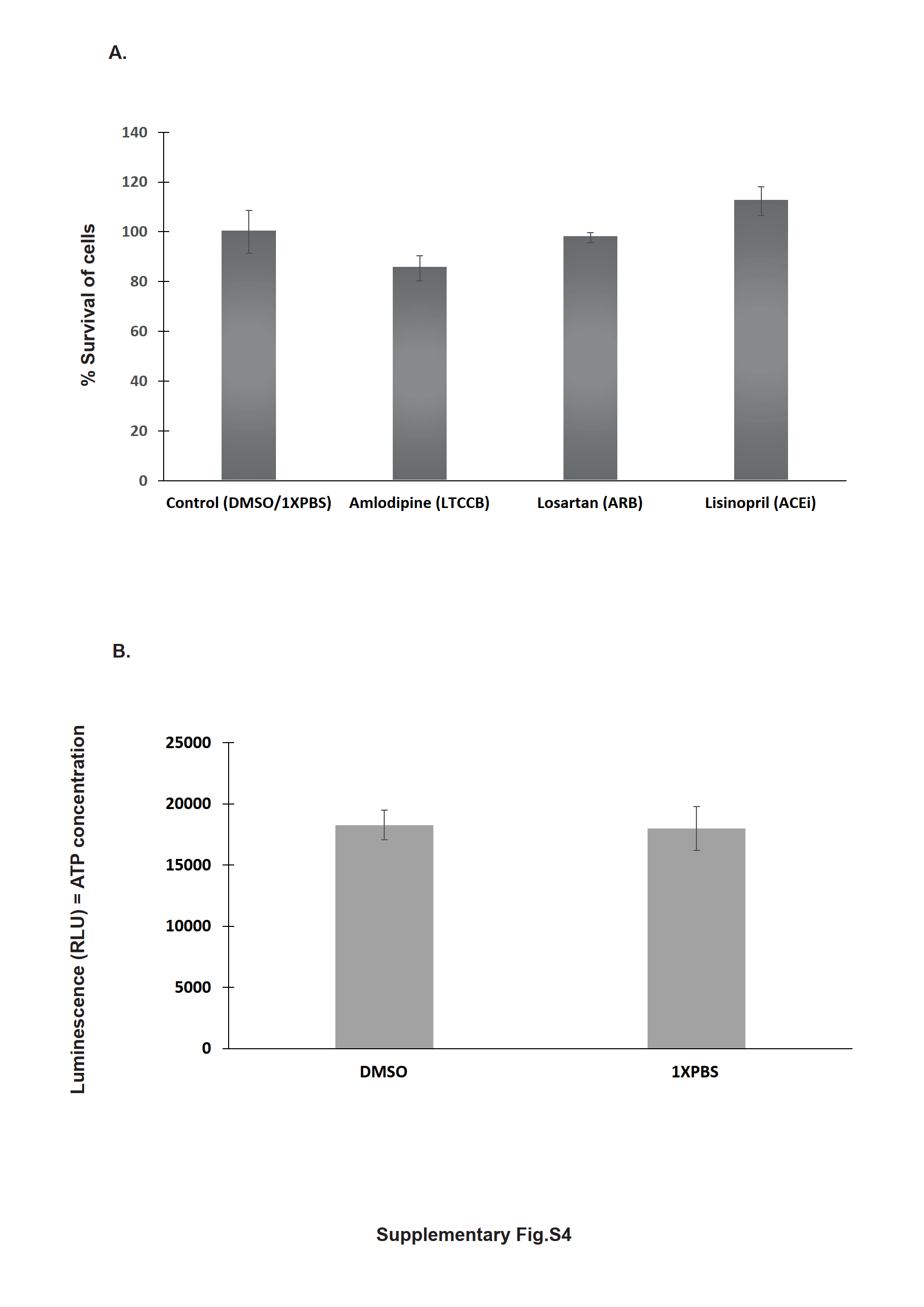

Supplement: S4 Fig — A. MIO-M1 cells viability determination. Survival of MIO-M1 cells at end of drug treatment (96 hours), based on intracellular ATP concentration. Survival mean percentages are calculated from two independent experiments. Vertical lines represent standard deviation. B. MIO-M1 cells viability, DMSO vs 1xPBS treatment. Luminescence [quantified as Relative Light Units (RLU)] based assay to measure intracellular ATP indicating general cell health. Our data show no statistically significant difference in the intracellular ATP concentration between DMSO and 1xPBS in MIO-M1 cells; 96 hours post treatment (End of experiment). Vertical lines represent standard deviation. (TIF) [file pone.0284364.s004.tif]
