# Supplementary material for: L-type calcium channel blocker increases VEGF concentrations in retinal cells and human serum
Source: PLoS One. 2023 Apr 13;18(4):e0284364. doi: 10.1371/journal.pone.0284364 (PMC10101440; doi:10.1371/journal.pone.0284364)
Supplement: S1 Appendix — List of FinnDiane study centers across Finland. (DOCX) [file pone.0284364.s007.docx]

**Supplementary Table 1:** Physicians and nurses at each of the FinnDiane centers participating in patient recruitment and characterization

**The Finnish Diabetic Nephropathy Study Center Physicians and nurses**

Anjalankoski Health Center S.Koivula, T.Uggeldahl

Central Finland Central Hospital, Jyväskylä T.Forslund, A.Halonen, A.Koistinen, P.Koskiaho, M.Laukkanen, J.Saltevo, M.Tiihonen

Central Hospital of Åland Islands, Mariehamn M.Forsen, H.Granlund, A.-C.Jonsson, B.Nyroos

Central Hospital of Kanta-Häme, Hämeenlinna P.Kinnunen, A.Orvola, T.Salonen, A.Vähänen

Central Hospital of Kymenlaakso, Kotka R.Paldanius, M.Riihelä, L.Ryysy

Central Hospital of Länsi-Pohja, Kemi H.Laukkanen, P.Nyländen, A.Sademies

Central Ostrobothnian Hospital District, Kokkola S.Anderson, B.Asplund, U.Byskata, P.Liedes, M.Kuusela, T.Virkkala

City of Espoo Health Center:

Espoonlahti A.Nikkola, E.Ritola

Tapiola M.Niska, H.Saarinen

Samaria E.Oukko-Ruponen, T.Virtanen

Viherlaakso A.Lyytinen

City of Helsinki Health Center:

Puistola H.Kari, T.Simonen

Suutarila A.Kaprio, J.Kärkkäinen, B.Rantaeskola

Töölö P.Kääriäinen, J.Haaga, A-L.Pietiläinen

City of Hyvinkää Health Center S.Klemetti, T.Nyandoto, E.Rontu, S.Satuli-Autere

City of Vantaa Health Center:

Korso R.Toivonen, H.Virtanen

Länsimäki R.Ahonen, M.Ivaska-Suomela, A.Jauhiainen

Martinlaakso M.Laine, T.Pellonpää, R.Puranen

Myyrmäki A.Airas, J.Laakso, K.Rautavaara

Rekola M.Erola, E.Jatkola

Tikkurila R.Lönnblad, A.Malm, J.Mäkelä, E.Rautamo

Heinola Health Center P.Hentunen, J.Lagerstam

Helsinki University Central Hospital, M.Feodoroff, D.Gordin, O.Heikkilä, K.Hietala, J.Fagerudd,

Department of Medicine M.Korolainen, L.Kyllönen, J.Kytö, S.Lindh,

Division of Nephrology K.Pettersson-Fernholm, M.Rosengård-Bärlund, A.Sandelin,

L.Thorn, J.Tuomikangas, T.Vesisenaho, J.Wadén

Herttoniemi Hospital, Helsinki V.Sipilä

Hospital of Lounais-Häme, Forssa T.Kalliomäki, J.Koskelainen, R.Nikkanen, N.Savolainen, H.Sulonen,

E.Valtonen

Hyvinkää Hospital L. Norvio, A.Hämäläinen

Iisalmi Hospital E.Toivanen

Jokilaakso Hospital, Jämsä A.Parta, I.Pirttiniemi

Jorvi Hospital, HUS, Espoo S.Aranko, S.Ervasti, R.Kauppinen-Mäkelin, A.Kuusisto, T.Leppälä, K.Nikkilä,

L.Pekkonen

Jyväskylä Health Center, Kyllö K.Nuorva, M.Tiihonen

Kainuu Central Hospital, Kajaani S.Jokelainen, K.Kananen, M.Karjalainen, P.Kemppainen, A-M.Mankinen,

A.Reponen, M.Sankari

Kerava Health Center H.Stuckey, P.Suominen

Kirkkonummi Health Center A.Lappalainen, M.Liimatainen, J.Santaholma

Kivelä Hospital, Helsinki A.Aimolahti, E.Huovinen

Koskela Hospital, Helsinki V.Ilkka, M.Lehtimäki

Kotka Health Center E.Pälikkö-Kontinen, A.Vanhanen

Kouvola Health Center E.Koskinen, T.Siitonen

Kuopio University Hospital E.Huttunen, R.Ikäheimo, P.Karhapää, P.Kekäläinen, M.Laakso, T.Lakka,

E.Lampainen, L.Moilanen, S. Tanskanen, L.Niskanen, U.Tuovinen,

I.Vauhkonen, E.Voutilainen

Kuusamo Health Center T.Kääriäinen, E.Isopoussu

Kuusankoski Hospital E.Kilkki, I.Koskinen, L.Riihelä

Laakso Hospital, Helsinki T.Meriläinen, P.Poukka, R.Savolainen, N.Uhlenius

Lahti City Hospital A.Mäkelä, M.Tanner

Lapland Central Hospital, Rovaniemi L.Hyvärinen, K.Lampela, S.Pöykkö, T.Rompasaari, S.Severinkangas, T.Tulokas

Lappeenranta Health Center P. Erola, L.Härkönen, P.Linkola, T.Pekkanen, I.Pulli, E.Repo

Lohja Hospital T.Granlund, K.Hietanen, M.Porrassalmi, M.Saari, T.Salonen, M.Tiikkainen,

Länsi-Uusimaa Hospital, Tammisaari I.-M.Jousmaa, J.Rinne

Loimaa Health Center A.Mäkelä, P.Eloranta

Malmi Hospital, Helsinki H.Lanki, S.Moilanen, M.Tilly-Kiesi

Mikkeli Central Hospital A.Gynther, R.Manninen, P.Nironen, M.Salminen, T.Vänttinen

Mänttä Regional Hospital I.Pirttiniemi, A-M.Hänninen

North Karelian Hospital, Joensuu U-M.Henttula, P.Kekäläinen, M.Pietarinen, A.Rissanen, M.Voutilainen

Nurmijärvi Health Center A.Burgos, K.Urtamo

Oulaskangas Hospital, Oulainen E.Jokelainen, P-L.Jylkkä, E.Kaarlela, J.Vuolaspuro

Oulu Health Center L.Hiltunen, R.Häkkinen, S.Keinänen-Kiukaanniemi

Oulu University Hospital R.Ikäheimo

Päijät-Häme Central Hospital H.Haapamäki, A.Helanterä, S.Hämäläinen, V.Ilvesmäki, H.Miettinen

Palokka Health Center P.Sopanen, L.Welling

Pieksämäki Hospital V.Sevtsenko, M.Tamminen

Pietarsaari Hospital M-L.Holmbäck, B.Isomaa, L.Sarelin

Pori City Hospital P.Ahonen, P.Merisalo, E.Muurinen, K.Sävelä

Porvoo Hospital M.Kallio, B.Rask, S.Rämö

Raahe Hospital A.Holma, M.Honkala, A.Tuomivaara, R.Vainionpää

Rauma Hospital K.Laine, K.Saarinen, T.Salminen

Riihimäki Hospital P.Aalto, E.Immonen, L.Juurinen

Salo Hospital A.Alanko, J.Lapinleimu, P.Rautio, M.Virtanen

Satakunta Central Hospital, Pori M.Asola, M.Juhola, P.Kunelius, M.-L.Lahdenmäki, P.Pääkkönen, M.Rautavirta

Savonlinna Central Hospital T.Pulli, P.Sallinen, M.Taskinen, E.Tolvanen, T.Tuominen, H.Valtonen,

A.Vartia, S-L. Viitanen

Seinäjoki Central Hospital O.Antila, E.Korpi-Hyövälti, T.Latvala, E.Leijala, T.Leikkari, M.Punkari,

N.Rantamäki, H.Vähävuori

South Karelia Central Hospital, T.Ensala, E.Hussi, R.Härkönen, U.Nyholm, J.Toivanen

Lappeenranta

Tampere Health Center A.Vaden, P.Alarotu, E.Kujansuu, H.Kirkkopelto-Jokinen, M.Helin,

S.Gummerus, L.Calonius, T.Niskanen, T.Kaitala, T.Vatanen

Tampere University Hospital P. Hannula, I.Ala-Houhala, R.Kannisto, T.Kuningas, P.Lampinen, M.Määttä,

H.Oksala, T.Oksanen, A.Putila, H.Saha, K.Salonen, H.Tauriainen, S.Tulokas

Tiirismaa Health Center, Hollola T.Kivelä, L.Petlin, L.Savolainen

Turku Health Center A.Artukka, I.Hämäläinen, L.Lehtinen, E.Pyysalo, H.Virtamo, M.Viinikkala,

M.Vähätalo

Turku University Central Hospital K.Breitholz, R.Eskola, K.Metsärinne, U.Pietilä, P.Saarinen, R.Tuominen,

S.Äyräpää

Vaajakoski Health Center K.Mäkinen, P.Sopanen

Valkeakoski Regional Hospital S.Ojanen, E.Valtonen, H.Ylönen, M.Rautiainen,T.Immonen

Vammala Regional Hospital I.Isomäki, R.Kroneld, L.Mustaniemi, M.Tapiolinna-Mäkelä

Vaasa Central Hospital S.Bergkulla, U.Hautamäki, V-A.Myllyniemi, I.Rusk
